# Strategy for lung parenchyma-sparing bronchial resection: a case series report

**DOI:** 10.1093/icvts/ivac166

**Published:** 2022-06-14

**Authors:** Akihiro Ohsumi, Hidenao Kayawake, Yoshito Yamada, Satona Tanaka, Yojiro Yutaka, Daisuke Nakajima, Masatsugu Hamaji, Toshi Menju, Hiroshi Date

**Affiliations:** Department of Thoracic Surgery, Kyoto University Hospital, Kyoto, Japan; Department of Thoracic Surgery, Kyoto University Hospital, Kyoto, Japan; Department of Thoracic Surgery, Kyoto University Hospital, Kyoto, Japan; Department of Thoracic Surgery, Kyoto University Hospital, Kyoto, Japan; Department of Thoracic Surgery, Kyoto University Hospital, Kyoto, Japan; Department of Thoracic Surgery, Kyoto University Hospital, Kyoto, Japan; Department of Thoracic Surgery, Kyoto University Hospital, Kyoto, Japan; Department of Thoracic Surgery, Kyoto University Hospital, Kyoto, Japan; Department of Thoracic Surgery, Kyoto University Hospital, Kyoto, Japan

**Keywords:** Lung cancer, Bronchial sleeve resection, Bronchoplasty, Lung parenchyma-sparing

## Abstract

**Figure:**
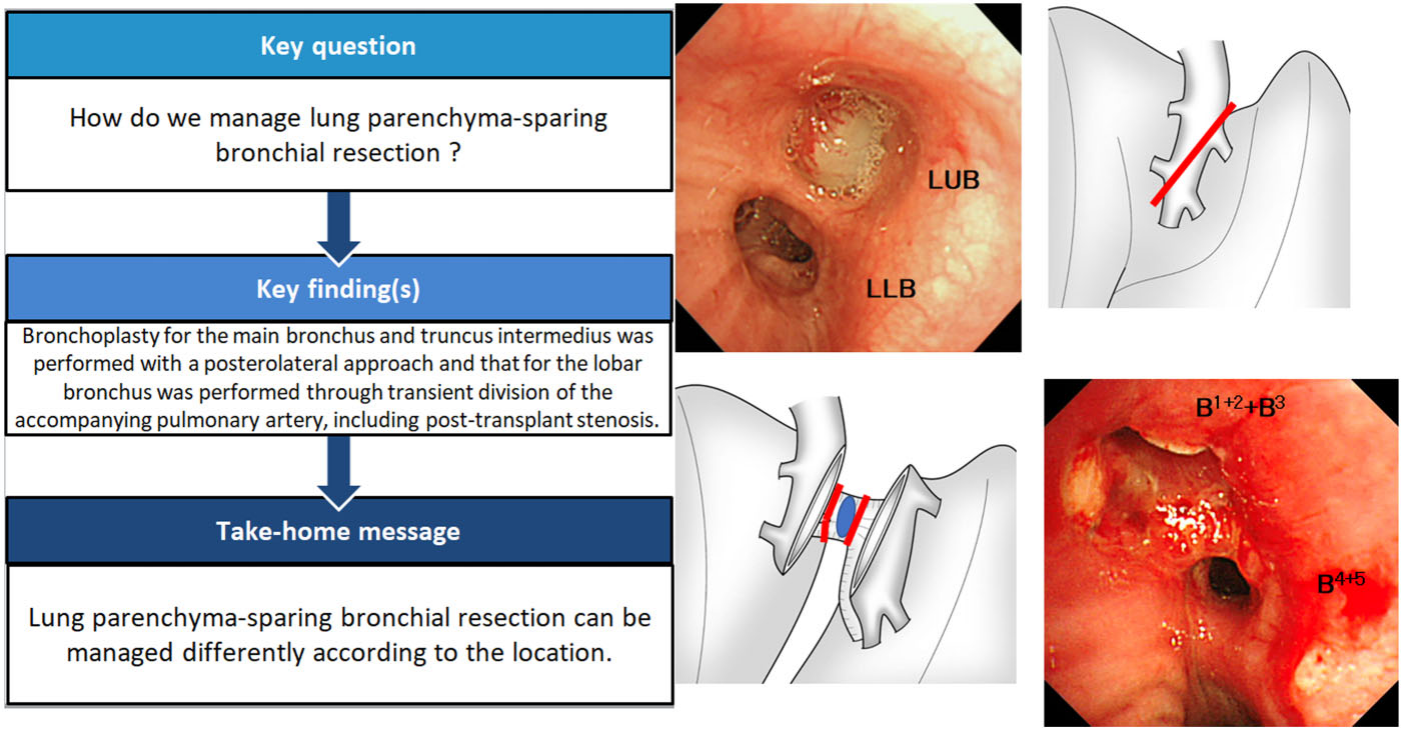

Lung parenchyma-sparing bronchial resection is uncommon, and the operative procedure depends on the cause and location of the stenosis. We present 6 cases and discuss the different surgical strategies for sleeve resection of the central airway without lung resection. Bronchoplasty for the main bronchus and truncus intermedius was performed with a posterolateral approach. We resected the right main bronchus including the right lateral wall of the lower trachea and half of the carina obliquely and performed an anastomosis. The tumour in the left lobar bronchus was exposed and removed by transient division of the accompanying pulmonary artery. Although post-transplant stenosis and malacia can pose a challenge, bronchoplasty can be used as a definitive treatment in experienced centres.

## INTRODUCTION

Sleeve lobectomy for lung cancer is an established technique and has been widely used with safety. However, sleeve resection of the bronchus sparing the lung parenchyma is an uncommon procedure, because it is limited to benign tumours, low-grade malignancies originating from the central airway or mechanical stenosis or kinking without a tumour, and it depends on the location of the stenotic lesion. We present 6 cases to discuss the surgical strategy for sleeve resection of the central airway without lung resection.

## PATIENTS AND METHODS

### Ethics statement

Ethical approval for this study was obtained from the review board of the ethics committee of Kyoto University Graduate School and Faculty of Medicine (No. R2504, June 22, 2020). Oral and written consent were obtained from all patients.

## Case 1

**Figure 1:**
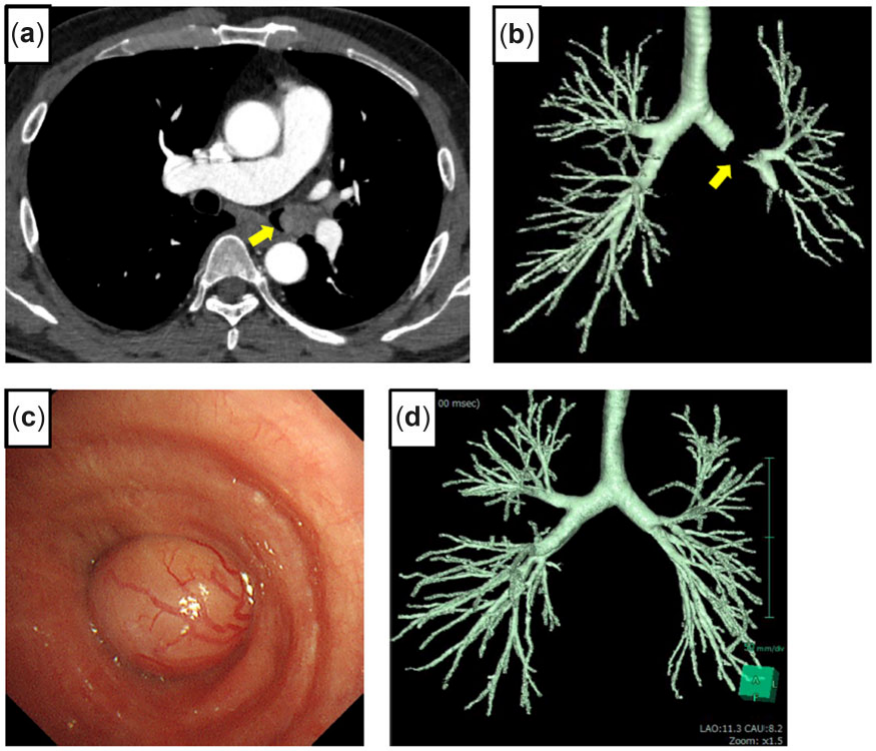
Chest computed tomography shows a tumour occupying the lower left main bronchus (arrow points to the tumour) (**a**). A 3-dimensional computed tomography scan shows almost total obstruction by the tumour (arrow points to the obstruction) (**b**). Bronchoscopy shows a tumour obstructing the lower left main bronchus (**c**). Postoperative 3-dimensional computed tomography shows airway lumen patency (**d**).

A 55-year-old male with diabetes mellitus had haemosputum and was found to have a tumour obstructing the left main bronchus (LMB) on computed tomography (CT) of the chest (Fig. 1a). To further evaluate the stenosis, we reviewed the 3-dimensional (3D) CT reconstruction images with SYNAPSE VINCENT (Fujifilm, Tokyo, Japan) software (Fig. 1b). The patient had obstructive pneumonia and was treated with antibiotics. Bronchoscopy showed a bronchial tumour obstructing the LMB; the tumour was not biopsied due to hypervascularity on the surface (Fig. 1c). A benign or low-grade malignant tumour was suspected. We opened the left chest with a posterolateral incision, sparing the serratus anterior muscle, through the fifth intercostal space. The LMB was dissected and removed; the proximal and distal sides were just above the tumour and the bifurcation of the upper and lower bronchi, respectively. The tumour was diagnosed as adenoid cystic carcinoma, and the bronchial stump had microscopic invasion of tumour cells at both the proximal and distal sides. Moreover, additional resection of the distal side of the LMB showed positive results; therefore, total resection of the bronchus with cancer cells was considered impossible. An anastomosis was performed with continuous suture of the mediastinal side and interrupted suture of the other part using 4–0 polydioxanone (PDS) covered with pedicled pericardial fat tissue. Radiation therapy was performed for the complete central airway of the left side (total, 66 Gy). Chest CT scans after the operation revealed complete opening of the anastomosis (Fig. 1d); the patient has not shown any symptoms after 21 months.

## Case 2

**Figure 2:**
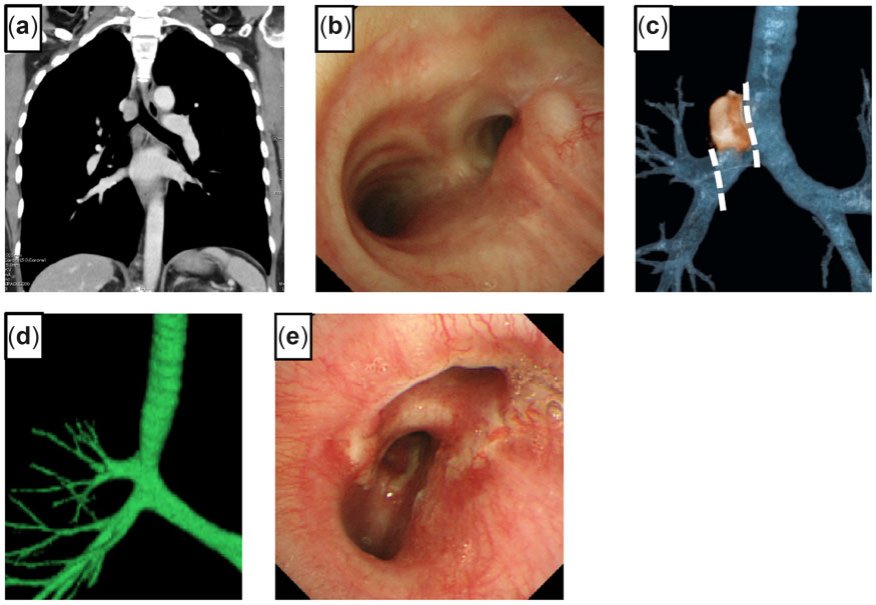
Chest computed tomography in a coronal view shows a tumour around the proximal right main bronchus (**a**). Bronchoscopy shows the membranous part of the right lateral wall of the lower trachea pressed by a tumour (**b**). The bronchus occupied by a tumour was divided obliquely at the proximal and distal sides (**c**). A 3-dimensional computed tomography scan shows airway lumen patency of the anastomosis (**d**). Bronchoscopy 2 months after the operation shows healing of the anastomosis (truncus intermedius).

A 37-year-old female had a neurinoma arising from the right lateral wall of the lower trachea invading the right main bronchus (RMB), the length of which was 2.3 cm (Fig. 2a, b). Seven years ago, she had dyspnea. The tumour was cauterized by flexible fibre-optic bronchoscopy. However, it subsequently returned; she was referred to our hospital. With posterolateral thoracotomy, the azygos vein was divided, and the trachea and RMB were taped. A right hilar release was performed by dissection of the pulmonary ligament and a U-shaped pericardial incision below the lower pulmonary vein (PV). Under bronchoscopic guidance, the lower tracheal circumference up to 3 rings from the carina and the RMB were resected obliquely on the proximal side (Fig. 2c). The length of the resected specimen was about 3 cm, and the top of the defective trachea was closed directly. The anastomosis was performed with continuous suture for the mediastinal side and interrupted suture for the other part using 4–0 PDS covered with pedicled pericardial fat (Video 1). Bronchoscopy and 3D-CT showed normal healing of the anastomosis without stenosis after 2 months (Fig. 2d, e).

## Case 3

**Figure 3:**
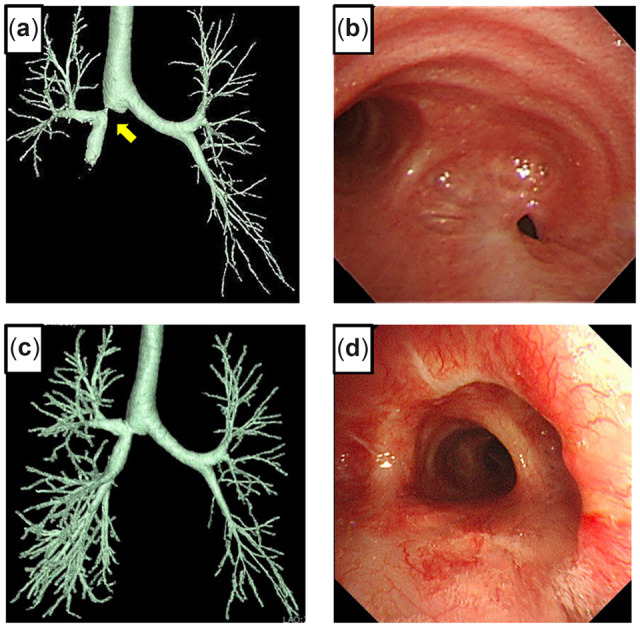
Three-dimensional computed tomography shows severe stricture (arrow points to the stricture) (**a**). Bronchoscopy shows a pinhole with granulated tissue at the orifice of the right main bronchus (**b**). A 3-dimensional scan shows decent passage of the anastomosis (**c**). Bronchoscopy 3 months after surgery shows healing and lumen patency of the anastomosis (**d**).

The details of this case with a video have been described previously (1). A 34-year-old male had a motor vehicle accident at the age of 21. He recently had recurrent obstructive pneumonia. The chest CT showed ground-glass opacities with consolidation in the right middle and lower lobes due to stenosis of the RMB. The 3D-CT scan showed severe stenosis at the RMB orifice (Fig. 3a). Using fibre-optic bronchoscopy, we identified almost complete stenosis caused by a granulomatous lesion with a pinhole by (Fig. 3b). The patient was referred to our hospital for surgical intervention. We opened the right chest using a posterolateral incision that spared the serratus anterior muscle through the fourth intercostal space. The RMB was opened at the proximal side; however, the stenotic lesion remained. We further resected half of the tracheal carina and the lower part of the trachea. On the distal side, the main bronchus was removed to the smallest extent possible (about 2 cm). An anastomosis was performed with continuous suture of the mediastinal side and interrupted suture of the other part using 4–0 PDS covered with pedicled pericardial fat tissue. The patient was discharged without any postoperative complications. The pathological analysis of the resected specimen showed the presence of granulation tissue with a focal fibrosis lesion. Follow-up chest CT (Fig. 3c) and bronchoscopy (Fig. 3d) 3 months after surgery showed healing of the anastomosis with luminal patency.

## Case 4

**Figure 4:**
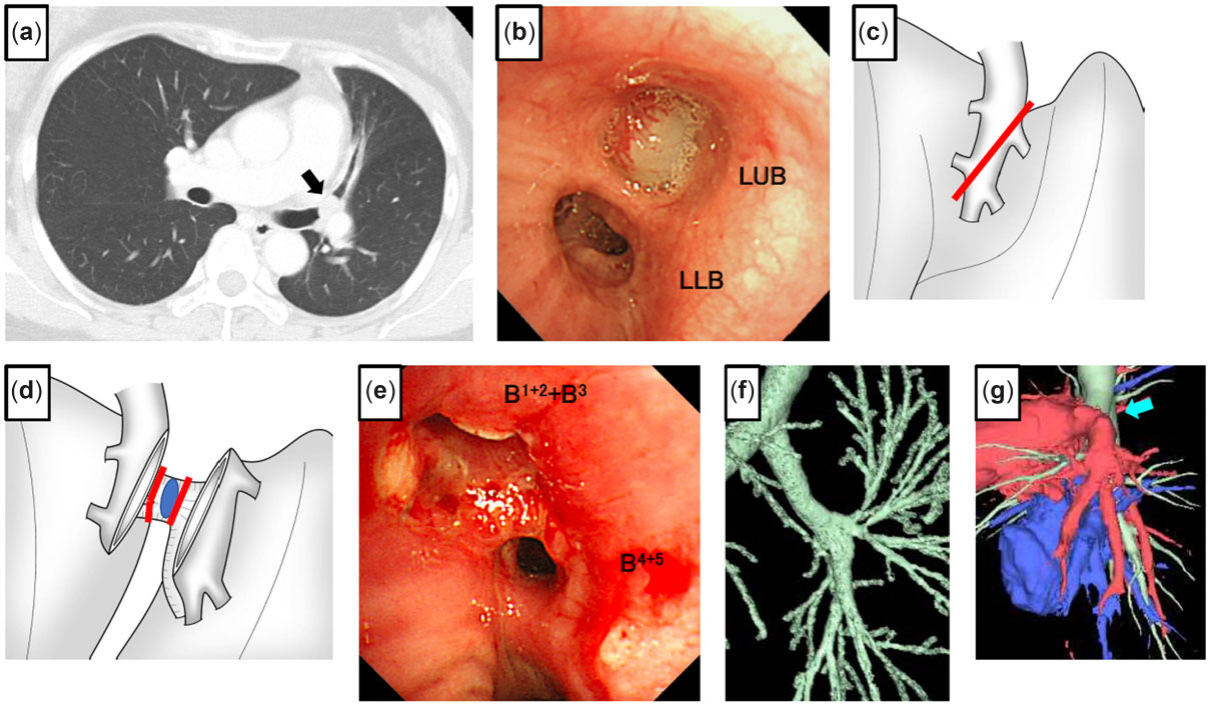
Chest computed tomography shows a tumour occupying the left upper bronchus (LUB); the arrow points to a tumour (**a**). Bronchoscopy before surgery shows a tumour obstructing the LUB (LLL: left lower bronchus) (**b**). The interlobar pulmonary artery was divided obliquely to expose the LUB (**c**). The LUB was exposed and removed with the tumour (**d**).

A 50-year-old female had atelectasis of the left lung at the annual medical check-up. Chest CT and bronchoscopy showed a tumour obstructing the orifice of the left upper bronchus, which was later diagnosed as a leiomyoma (Fig. 4a, b). She was referred to our institute for surgical resection. The left chest was opened through the fifth intercostal space with a posterolateral incision sparing the serratus anterior muscle. Each arterial branch of the pulmonary artery (PA) of the left upper lobe and superior and inferior PV was dissected and secured. After systemic heparinization, the main PA, superior segmental artery, basal artery and superior PV were clamped, and the interlobar PA was divided obliquely (Fig. 4c). The left upper bronchus was exposed, and sleeve resection was performed including the tumour (Fig. 4d). The distal margin of the lingular bronchus was insufficient; therefore, the bronchi of the superior and lingular division were further divided, and the additional lingular bronchus was removed. The superior and lingular bronchi were anastomosed in a side-to-side fashion and later anastomosed to the proximal orifice of the left upper bronchus. Lastly, the divided interlobar PA was re-anastomosed and reperfused (Video 2). Postoperative bronchoscopy showed satisfactory healing (Fig. 4e), and 3D-CT identified no stenosis of the bronchial and PA anastomosis (Fig. 4f, g).

## Case 5

**Figure 5:**
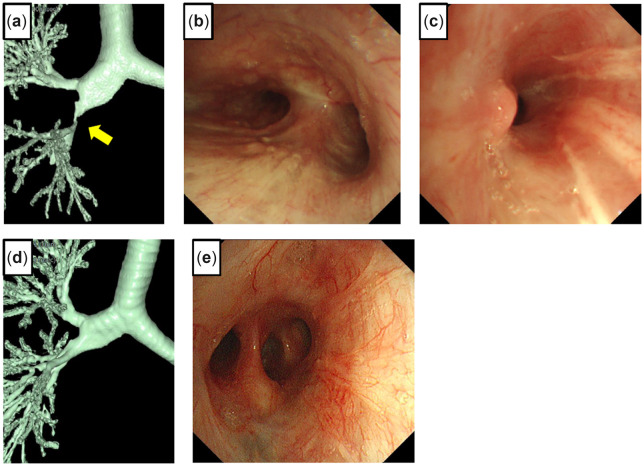
Three-dimensional computed tomography 6 months after a lung transplant shows severe stricture of the truncus intermedius (TIM) (**a**). Bronchoscopy 6 months after the lung transplant shows healing with complete lumen patency of the right main bronchial anastomosis (**b**) and severe stricture at the distal side of the TIM **(c**). A 3-dimensional computed tomography scan 2 years after TIM reconstruction shows lumen patency (**d**). Bronchoscopy 2 years after reconstruction shows healing of the anastomosis (**e**).

A 51-year-old male underwent a double lung transplant (LTx) from a cadaveric donor for familial pulmonary arterial hypertension; the post-transplant course was uneventful. The 3D-CT scan showed severe stenosis at the end of the truncus intermedius (TIM) after 6 months (Fig. 5a). Bronchoscopic findings showed normal healing of the right anastomosis (Fig. 5b) and a severe stenotic lesion at the distal side of the TIM (Fig. 5c). Stent placement was unsuccessful; therefore, a rethoracotomy was performed. The right chest was opened through the fifth intercostal space with a posterolateral incision, and the lung was dissected from the chest wall. The superior segmental artery (A^6^) and posterior ascending artery (A^2^) were dissected and secured between the upper and lower lobes; however, the TIM was difficult to encircle due to adhesions to the surrounding tissue; therefore, it was opened under bronchoscopic guidance. The bronchial wall was fragile but hypertrophic, and the stenotic lesion was removed. For a tension-free anastomosis, the middle and lower lobes were dissected from the chest wall, diaphragm and pericardium. The bronchial proximal and distal orifices without stenotic lesions were anastomosed with continuous suture of the mediastinal side and interrupted suture of the other part using 4–0 PDS covered with an omental flap. Bronchoscopy and 3D-CT showed healing of the anastomosis without stenosis after 2 years (Fig. 5d, e).

## Case 6

**Figure 6:**
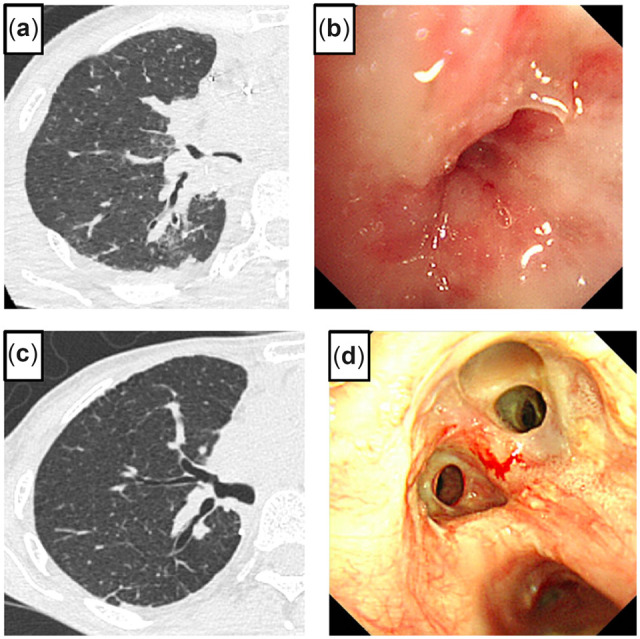
Chest computed tomography shows kinking of the anastomosed right upper bronchus (RUB) (**a**). Bronchoscopy before rethoracotomy shows narrowing of the anastomosed RUB (**b**). A chest computed tomography scan shows passage of the re-anastomosed RUB (**c)**. Bronchoscopy shows healing and passage of the re-anastomosed RUB (**d**).

A 43-year-old female underwent a double LTx from a cadaveric donor for idiopathic interstitial pneumonia by clamshell incision. The donor grafts were larger than the patient’s chest cavity. To downsize the graft, the donor’s right lower lobe was removed at the back table, and the right upper and middle lobes were anastomosed to the recipient’s upper bronchus and TIM. Moreover, only the left lower lobe was transplanted into the left chest cavity. After reperfusion, she developed primary graft dysfunction requiring extracorporeal membrane oxygenation support. A chest CT scan and bronchoscopy showed severe stenosis of the anastomosed right upper bronchus (RUB) (Fig. 6a, b); rethoracotomy was performed 4 days later. The right main PA was clamped between the ascending aorta and superior vena cava, and the left atrium was clamped. The PA anastomosis was re-opened to reach the right upper bronchial anastomosis. The proximal and distal sides of the right upper bronchi were trimmed and re-anastomosed. The re-opened PA was anastomosed again, and the lung graft was ventilated and reperfused; then, the extracorporeal membrane oxygenation was successfully removed. A chest CT scan and bronchoscopy showed satisfactory healing with no stenosis (Fig. 6c, d).

## DISCUSSION

We used several surgical strategies to perform lung parenchyma-sparing bronchial resection for bronchial stenosis in different situations. Previous reports have proposed different kinds of bronchoplasty: resection of the carina (2), tracheobronchial bifurcation (3, 4), right main stem and TIM (2, 5–7), LMB (2, 5, 8, 9), left secondary carina (10, 11) and lobar bronchus and bronchial corner (2). Here, we focused mainly on the surgical approach for dealing with the lesions. When determining the indications for lung parenchyma-sparing bronchial resection, benign diseases (post-traumatic lesions, benign tumours and low-grade malignancies including adenoid cystic carcinoma, carcinoid tumour and mucoepidermoid tumour) should be considered suitable for lung parenchyma-sparing bronchial resection, which is in accordance with previous reports (4).

### Oblique bronchial anastomosis

In cases of sleeve bronchial resection with lung parenchyma such as sleeve lobectomy or segmentectomy, the PA and PV are usually divided first followed by lymph node dissection. The bronchial trees are then released sufficiently; finally, the bronchus is divided and anastomosed. However, bronchial resection sparing the lung parenchyma does not always need vessel division and lymph node dissection; sometimes it needs various releases for a tension-free anastomosis. Therefore, if the resection is limited to the central airway, an approach to the vessels is not required. Resection of the main stem could be possible through a smaller incision preserving the serratus anterior from only the posterior view of the hilum (cases 1 and 3) or by the thoracoscopic approach. However, in cases of tight adhesion with the surrounding tissue due to traumatic injury (case 3) or rethoracotomy (case 5), the securing bronchus is extremely tough; therefore, vessel preparation and securing are required for safety. Moreover, encircling the bronchus is challenging; it would be easier to divide the bronchus first and dissect it from the surrounding tissue sufficiently and then anastomose it without tension. The approach to the lesion depends on the location. The RMB and TIM are reachable from the posterior hilum (cases 1 and 3). When the lesion in the RMB extends to the right lateral wall of the lower trachea, it is possible to divide the lower trachea and TIM in an oblique fashion and anastomose both stumps (cases 2 and 3). This technique can be applied to locally advanced lung cancer, and an auto-lung transplant is one of the solutions, as previously reported (12). Mantovani *et al.* suggested different approaches for bronchial anastomosis in proximal and distal lesions at the LMB because it is longer than the RMB. A posterolateral incision was performed for distal lesions such as in case 1, whereas the upper part of the median sternotomy was performed for the proximal lesions, which were exposed both transpericardially after adequate mobilization of the aortic arch and transpleurally with ample dissection of the left main artery (9).

### Pulmonary artery division and re-anastomosis

All lobar bronchi are surrounded by PA and PV branches except the RUB: Only the RUB can be exposed without mobilization of the PA or PV from behind. Resection and reconstruction of the lobar bronchi, except RUB without lung resection, will be potentiated only in cases of initial surgery (case 4) or just after surgery (case 6). To our knowledge, no researchers have reported this strategy of bronchial sleeve resection following division of the accompanying PA. The lobar bronchus is short and the tumour-free margin is sometimes inadequate; therefore, additional resection of segmental bifurcation and branches followed by plasty of *de novo* segmental bifurcation is an acceptable solution (case 4).

### Bronchial stenosis after a lung transplant

The International Society of Heart and Lung Transplantation published a consensus statement on airway complications and described 2 types of stenosis: central airway stenosis and distal airway stenosis. Distal airway stenosis mostly occurs at the TIM and has been reported in about 2% of total cases (13). Various treatment strategies have been introduced: balloon dilation, endobronchial stent placement, laser therapy, electrocautery, argon plasma coagulation and cryotherapy. In rare cases, surgery may be utilized, and ultimately some patients undergo a retransplant (13). Furthermore, Mahajan *et al.* stated that a small subset of refractory bronchial stenosis cases required surgical intervention including anastomotic reconstruction or sleeve resection with or without lobar resection, but rarely a retransplant (14). At our institute, to prevent dehiscence, stenosis or malacia at TIM, the RUB is occasionally anastomosed to the recipient’s RUB stump and the middle and/or lower bronchi, to the TIM stump (case 6), when risk factors are noted in a donor or recipient. We considered bronchoplasty as a promising solution for stenosis or malacia of the central airway even after LTx (case 5). Though limited soon after transplant, peripheral airway reconstruction is possible by dividing the surrounding PA (case 6). Lari *et al.* reported a modified Montgomery T-tube technique for stenosis or malacia of TIM following an LTx (15). Another study by Faccioli *et al.* has shown the outcome of surgical management of post-transplant bronchial stenoses in a cohort of 35 patients with post-transplant stenoses (6 underwent bronchoplasty with or without lobectomies and 1 required a retransplant) (16). The surgical approach for post-transplant bronchial stenoses is challenging but beneficial and can lead to a definitive solution; however, it should be performed in experienced centres.

## CONCLUSIONS

Bronchoplasty of the main bronchus and TIM can be performed with a posterior surgical approach. Tumours invading the right lateral wall of the lower trachea can be resected obliquely and anastomosed with the TIM trimmed obliquely. Tumours in the lobar bronchus can be exposed and removed by transient division of the accompanying PA. Although post-transplant stenosis and malacia are challenging, bronchoplasty can be considered a definitive treatment in experienced centres.

## Conflict of Interest Statement

The authors have nothing to disclose with regard to commercial support.

## Data availability

All relevant data are within the manuscript and its supporting information files.
